# Association of hypertension with physical factors of wrist pulse waves using a computational approach: a pilot study

**DOI:** 10.1186/s12906-015-0756-7

**Published:** 2015-07-11

**Authors:** Bum Ju Lee, Young Ju Jeon, Boncho Ku, Jaeuk U. Kim, Jang-Han Bae, Jong Yeol Kim

**Affiliations:** KM Fundamental Research Division, Korea Institute of Oriental Medicine, 1672 Yuseongdae-ro, Yuseong-gu, Deajeon 305-811 Republic of Korea

**Keywords:** Wrist pulse wave, Hypertension, Physical factors, Computational approach, Quantification, Objectification, Traditional Chinese Medicine (TCM), Traditional Korean Medicine (TKM)

## Abstract

**Background:**

The objectives of this pilot study were to examine the association between hypertension and physical factors of wrist pulse waves to avoid subjective diagnoses in Traditional Chinese Medicine (TCM) and Traditional Korean Medicine (TKM). An additional objective was to assess the predictive power of individual and combined physical factors in order to identify the degree of agreement between diagnosis accuracies using physical factors and using a sphygmomanometer in the prediction of hypertension.

**Methods:**

In total, 393 women aged 46 to 73 years participated in this study. Logistic regression (LR) and a naïve Bayes algorithm (NB) were used to assess statistically significant differences and the predictive power of hypertension, and a wrapper-based machine learning method was used to evaluate the predictive power of combinations of physical factors.

**Results:**

In both wrists, L-PPI and R-PPI (maximum pulse amplitudes in the left Gwan and right Gwan) were the factors most strongly associated with hypertension after adjusting for age and body mass index (*p* = <0.001, odds ratio (OR) = 2.006 on the left and *p* = <0.001, OR = 2.504 on the right), and the best predictors (NB-AUC = 0.692, LR-AUC = 0.7 on the left and NB-AUC = 0.759, LR-AUC = 0.763 on the right). Analyses of both individual and combined physical factors revealed that the predictive power of the physical factors in the right wrist was higher than for the left wrist. The predictive powers of the combined physical factors were higher than those of the best single predictors in both the left and right wrists.

**Conclusion:**

We suggested new physical factors related to the sum of the area on the particular region of pulse waves in both wrists. L-PPI and R-PPI among all variables used in this study were good indicators of hypertension. Our findings support the quantification and objectification of pulse patterns and disease in TCM and TKM for complementary and alternative medicine.

## Background

Pulse diagnosis is one of the most important fields in Oriental medicine, including Traditional Chinese Medicine (TCM) and Traditional Korean Medicine (TKM), and has become a popular topic of research [1–7]. In pulse diagnosis in TCM and TKM, normal and abnormal pulse patterns related to organs such as the lungs, stomach, and heart are identified by palpation with the fingertips, indicating that the characteristics and variation of the pulse waves are evident in the frequency and time domains [7, 8]. In Oriental medicine, pulse diagnosis and patterns are dependent on the subjective judgment of Oriental medical doctors, and the description of pulse conditions is occasionally ambiguous.

Until recently, it was difficult to obtain reasonable and reliable information about wrist pulse waves in the time or frequency domains using the fingertips [8]. The diagnosis of normal or abnormal pulse patterns depends very much on the experience or intuition of the Oriental physician. Subjective pulse diagnosis requires a long learning period and is difficult to learn due to the lack of standardization and objectification of the wrist pulse and patterns and the association between pulse patterns and organs or diseases [7, 9–11]. Therefore, many TCM and TKM studies have sought to standardize the pulse-taking approach [2, 11, 12], the quantification of pulse patterns [2, 11, 13], and the association between pulse patterns and organs or particular diseases [2, 3, 11, 14], as well as to develop pulse wave detection devices [7, 15].

To standardize and quantify wrist pulse diagnosis and the measurement of wrist pulse waves, most studies have focused on the time and frequency domains to identify important physical determinants in the pulse waves. The time and frequency domains are often used to study diseases in both Western and Oriental medicine [2]. The physical factors of pulse waves in the time and frequency domains have been strongly associated with certain diseases and components such as depth, width, strength, length, smoothness, and stiffness in TCM [2, 9, 16–19]. Recent studies have sought to identify scientific evidence of wrist pulse patterns and disease using computational approaches [5, 7, 10, 20, 21]. The physical features or pulse patterns of the wrists have been associated with acute appendicitis [20], gastritis and cholecystitis [11], and duodenal bulb ulcers and pancreatitis [21]. Other studies have focused on the association between the wrist pulse wave and blood pressure (BP) [3–6]. These studies on the pulse condition based on the time domain have contributed to pulse diagnosis quantification because physical factors from the pulse waves support the physiological significance of the pulse [2].

The objective of this pilot study was to examine the association of hypertension with the physical factors from wrist pulse waves. An additional objective was to assess the predictive power of individual and combined physical factors for the prediction of hypertension in order to identify the degree of agreement between diagnosis accuracies using physical factors from the pulse wave and using a sphygmomanometer. The results of this study will be important for the quantification, objectification, and standardization of wrist pulse diagnosis and the association of pulse waves with organs or diseases [7, 9–11].

## Methods

### Subjects

In total, 393 Korean women aged 46 to 73 years participated in this study, and demographic data such as age, body mass index (BMI), and systolic and diastolic BPs were recorded for all participants. All assessments were conducted at the Oriental Hospital of Daejeon University in Cheonan City in the Republic of Korea between July 2012 and April 2013. The Institutional Review Board of the Korea Institute of Oriental Medicine (KIOM) approved the study (I0906-01-02), and written informed consent was obtained from all participants.

### Wrist pulse measurement

**Fig. 1:**
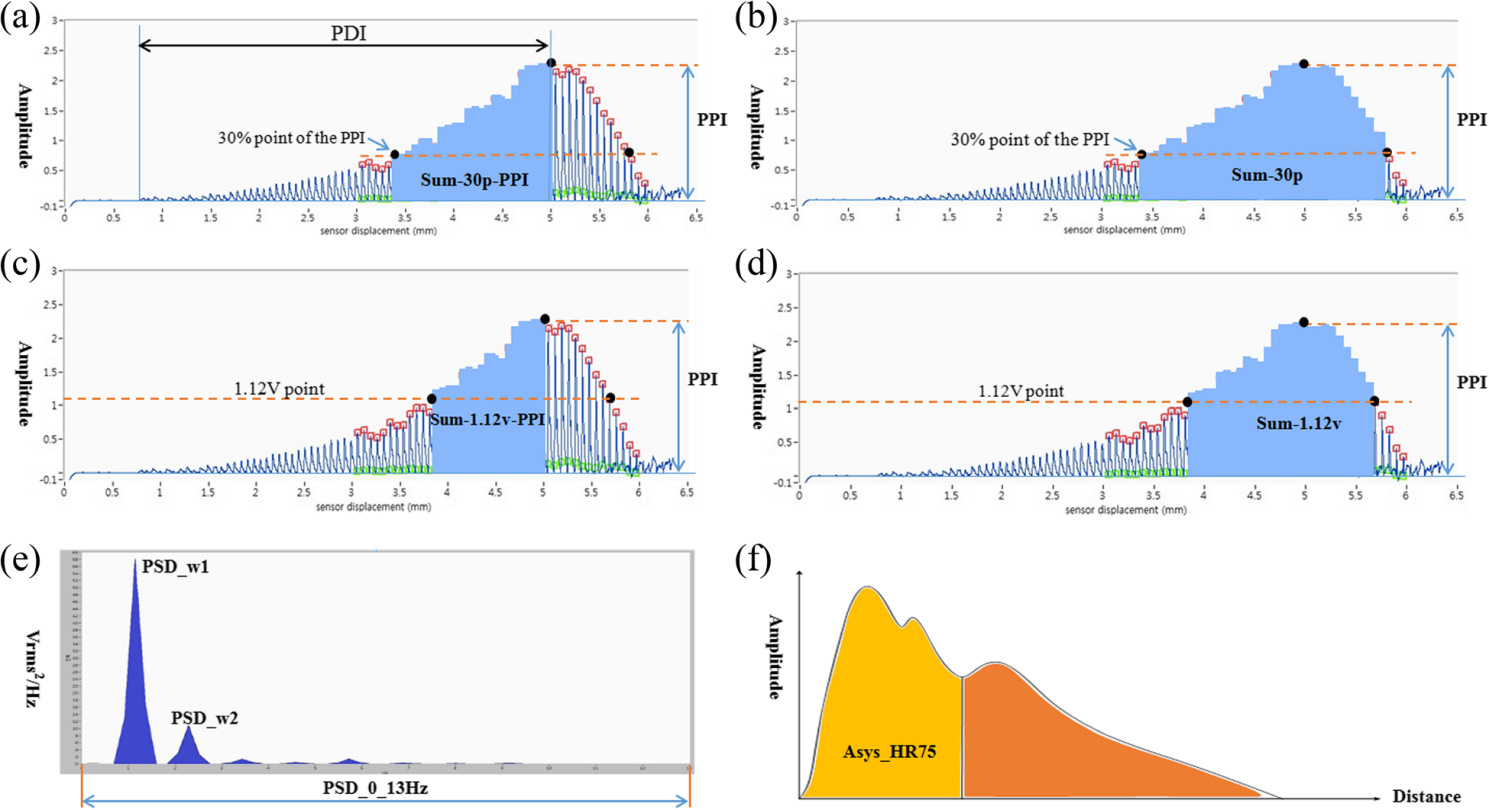
Detailed description of physical factors in pulse waves. **a** Sum-30p-PPI: sum of the pulse wave areas of the region from the 30 % point of PPI to PPI. **b** Sum-30p: sum of the pulse wave areas of the region of the pulse waves with amplitudes higher than 30 % of PPI. **c** Sum-1.12v-PPI: sum of the pulse wave areas of the region from 1.12 V point to PPI (1.12v means detection voltage of the pulse in the KIOM pulse wave detector device). **d** Sum-1.12v: sum of the pulse wave areas of the region with pulse amplitudes higher than 1.12v. **e** PSD_w1: Power spectral density (PSD) at the first harmonic frequency from the acquired pulse wave. **f** Asys_HR75: Area of systolic period after normalization with HR75 (heart rate of 75 bpm) for comparison among various heart rates

Both pulse waves were measured using the KIOM pulse wave detector device developed by the Korea Institute of Oriental Medicine (KIOM) [16]. The pulse detection sensor in the pulse wave detector device comprises 7 piezoresistive units. The wrist pulse measurements were obtained with the participants sitting upright in a comfortable posture. The accurate position of Gwan among the three positions was measured by Oriental medical doctors. In this study, we focused only on the Gwan position for the association between hypertension and the physical factors from the wrist pulse wave because the Gwan position is the center among the three pulse positions. Wrist pulse signals in the Gwan position were measured by a well-trained operator. In the signal processing step, we considered the band-pass filter, the spline interpolation method, and fast Fourier transform analysis for the removal of baseline wander and noise generated by subtle motion and breathing. Detailed information regarding the pulse measurement and pulse wave detector device is provided in reference [16]. We extracted various physical factors from the pulse waves and physical factors, and a brief description is provided in Table 1. Figure 1 illustrates the physical factors of the pulse waves. For example, PPI represents the maximum pulse amplitude in the Gwan position in both the left and right wrists, and PSD-0-13 signifies the area of the power spectrum density (PSD) between 0 and 13 Hz in the Gwan position.

**Table 1:** Basic characteristics and brief descriptions of the physical factors used in this study

Variable	Normotension	Hypertension	Description (unit)
Subjects	341	52	Number of normal subjects and hypertensive subjects
HEIGHT	155.8 (5.284)	154.3 (4.334)	Height
WEIGHT	58.497 (8.075)	59.187 (8.385)	Weight
BMI	24.063 (2.909)	24.823 (3.052)	Body mass index
Age	56.9 (5.525)	59.154 (5.782)	Age
SBP	113.9 (11.95)	148.2 (11.4)	Systolic blood pressure
DBP	70.45 (7.993)	85.6 (8.132)	Diastolic blood pressure
L-PPI	3.101 (0.667)	3.666 (0.864)	Maximum pulse amplitude in the left Gwan (V: voltage)
L-PDI	6 (1.709)	6.005 (1.823)	Sensor displacement from the skin contact point to the location of maximum pulse pressure in the left Gwan (mm)
L-Sum-30p	85.93 (40.19)	99.55 (49.78)	Sum of the pulse wave areas of the region of the pulse waves with amplitudes higher than 30 % of PPI in the left Gwan (V · s)
L-Sum-30p-PPI	54.78 (25.61)	61.65 (29.59)	Sum of the pulse wave areas of the region between the 30 % point of PPI and PPI in the left Gwan (V · s)
L-Sum-1.12v	82.7 (41.23)	98.58 (51.61)	Sum of the pulse wave areas of the region with pulse amplitudes higher than 1.12 V in the left Gwan (V · s)
L-Sum-1.12v-PPI	51.54 (26.97)	60.68 (31.76)	Sum of the pulse wave areas of the region between pulse amplitudes higher than 1.12 V and PPI in the left Gwan (V · s)
L-Asys-HR75	43.02 (3.749)	42.86 (3.214)	Area of systolic period after normalization with HR75 in the left Gwan (V · s)
L-PSD-w1	43.63 (8.169)	47.67 (8.837)	Power spectral density (PSD) at the first harmonic frequency in the left Gwan (Vrms^2^/Hz)
L-PSD-w2	10.56 (2.002)	11.5 (2.091)	PSD at the second harmonic frequency in the left Gwan (Vrms^2^/Hz)
L-PSD-0-13Hz	21.41 (1.919)	22.36 (1.663)	Area of PSD between 0 and 13 Hz in the left Gwan (Vrms^2^)
R-PPI	3.142 (0.693)	3.979 (0.992)	Maximum pulse amplitude in the right Gwan (V: voltage)
R-PDI	5.909 (1.737)	6.394 (2.196)	Sensor displacement from skin contact point to the location of maximum pulse pressure in the right Gwan (mm)
R-Sum-30p	83.02 (35.14)	108.8 (48.24)	Sum of the pulse wave areas of the region of the pulse waves with amplitudes higher than 30 % of PPI in the right Gwan (V · s)
R-Sum-30p-PPI	52.58 (21.48)	70.68 (32.82)	Sum of the pulse wave areas of the region between the 30 % point of PPI and PPI in the right Gwan (V · s)
R-Sum-1.12v	79.92 (35.85)	108.7 (50.34)	Sum of the pulse wave areas of the region with pulse amplitudes higher than 1.12 V in the right Gwan (V · s)
R-Sum-1.12v-PPI	49.48 (22.27)	70.48 (35.33)	Sum of the pulse wave areas of the region between pulse amplitudes higher than 1.12 V and PPI in the right Gwan (V · s)
R-Asys-HR75	43.16 (4.031)	43.88 (3.981)	Area of systolic period after normalization with HR75 in the right Gwan (V · s)
R-PSD-w1	41.63 (8.362)	46.22 (8.837)	PSD at first harmonic frequency in the right Gwan (Vrms^2^/Hz)
R-PSD-w2	9.984 (1.972)	10.73 (2.149)	PSD at second harmonic frequency in the right Gwan (Vrms^2^/Hz)
R-PSD-0-13Hz	20.85 (2.212)	22.25 (2.359)	Area of PSD between 0 and 13 Hz in the right Gwan (Vrms^2^)

### Definition of the diagnosis of hypertension

For the diagnosis of normotension and hypertension, we considered the recommendations of the World Health Organization [WHO] [22], the 2003 and 2013 European Society of Hypertension-European Society of Cardiology Guidelines for the management of arterial hypertension [23, 24], and criteria provided in previous studies [25, 26]. Specifically, normotension was defined as systolic blood pressure (SBP) <140 mmHg and/or diastolic blood pressure (DBP) <90 mmHg, and hypertension was defined as SBP ≥140 mmHg and/or DBP ≥90 mmHg.

### Statistical analysis and prediction configuration

All analyses of significant differences were performed using SPSS 19 for Windows (SPSS, Inc., Chicago, IL, USA), and all analyses of predictive power were conducted in Weka (the Waikato Environment for Knowledge Analysis data mining tool) [27]. For statistical analyses of the single physical factors of wrist pulse waves, binary logistic regression (LR) was used to examine any significant differences between hypertensive and normotensive subject groups after applying a standardization transformation. In the analyses of the predictive power of the individual physical factors, we used two machine-learning algorithms, LR and the naïve Bayes algorithm (NB), to produce more reliable and accurate results.

To achieve greater predictive power for hypertension and to compare the powers of individual physical factors and combined physical factors, two machine-learning algorithms (LR and NB) and a wrapper-based feature selection technique with an exhaustive search were used. In addition, we used the least absolute shrinkage selection operator (LASSO) method for solving multicollinearity among variables [28]. Lasso is one of the solutions to multicollinearity among variables that were highly correlated [29]. This experiment supports an experimental method to find the optimal variable set based on machine learning. All prediction experiments for predictive power were conducted using 5-fold cross validation. In biological and medical studies, the area under the receiver operating characteristic curve (AUC) is widely used to examine predictive power. Therefore, the AUC value was considered the primary criterion for the comparison of all predictive powers of the individual and combined physical factors.

## Results

### Analysis of single physical factors

This study included 341 normotensive and 52 hypertensive subjects. The mean (standard deviation) SBP and DBP were 113.9 (11.95) and 70.45 (7.993), respectively, in normotensive subjects and 148.2 (11.4) and 85.6 (8.132), respectively, in hypertensive subjects. Twenty physical factors (10 in the left wrist and 10 in the right wrist) were used to examine the association between physical factors and hypertension and to predict hypertension. Most of the physical factors were significantly different between normotensive and hypertensive subjects.

Tables 2 and 3 present the results of the association of hypertension with the physical factors extracted from the pulse waves in both the left and right wrists and the predictive power for hypertension of each physical factor. In the left wrist (Table 2), L-PPI was most strongly associated with hypertension (*p* = <0.001, odds ratio (OR) = 2.141 [95 % confidence interval, 1.589-2.885]) and maintained the strongest association after adjusting for age and BMI (adjusted *p* = <0.001, adjusted OR = 2.006 [1.472-2.734]). L-PPI displayed the best predictive power (NB-AUC = 0.692, LR-AUC = 0.7). L-PSD-w1 (adjusted *p* = 0.002, adjusted OR = 1.638 [1.196-2.244], NB-AUC = 0.621, LR-AUC 0.626) and L-PSD-0-13Hz (adjusted *p* = 0.003, adjusted OR = 1.615 [1.183-2.205], NB-AUC = 0.648, LR-AUC = 0.651) were also useful predictors of hypertension because these factors were highly associated with hypertension. In the right wrist (Table 3), R-PPI (*p* = <0.001, OR = 2.648 [(.909-3.672]) exhibited the strongest association with hypertension. This association was not substantially altered even after adjusting for age and BMI (adjusted *p* = <0.001, adjusted OR = 2.504 [1.802-3.479]). R-PPI (NB-AUC = 0.759, LR-AUC = 0.763) was the best predictor of hypertension. These results indicate that the predictive power of the physical factors of the right wrist is generally higher than that of the left wrist. The single indicators with the best predictive power are L-PPI and R-PPI.

**Table 2:** Association of hypertension with physical factors and predictive powers of individual factors in the Gwan position of the left wrist

Variable	Crude		Adjustment for age and BMI		Predictive power	
	p	OR	p	OR	NB-AUC	LR-AUC
L-PPI	<0.001	2.141 (1.589-2.885)	<0.001	2.006 (1.472-2.734)	0.692	0.7
L-PDI	0.985	1.003 (0.749-1.343)	0.881	0.978 (0.728-1.313)	0.447	0.444
L-Sum-30p	0.031	1.335 (1.027-1.737)	0.054	1.301 (0.996-1.7)	0.557	0.569
L-Sum-30p-PPI	0.081	1.27 (0.971-1.66)	0.138	1.228 (0.936-1.611)	0.554	0.563
L-Sum-1.12v	0.015	1.382 (1.065-1.795)	0.031	1.341 (1.027-1.75)	0.581	0.583
L-Sum-1.12v-PPI	0.029	1.341 (1.03-1.746)	0.064	1.288 (0.985-1.684)	0.584	0.59
L-Asys-HR75	0.766	0.956 (0.713-1.282)	0.922	1.015 (0.756-1.362)	0.499	0.478
L-PSD-w1	0.001	1.67 (1.222-2.283)	0.002	1.638 (1.196-2.244)	0.621	0.626
L-PSD-w2	0.002	1.577 (1.179-2.108)	0.008	1.49 (1.111-2)	0.619	0.621
L-PSD-0-13Hz	0.001	1.664 (1.224-2.261)	0.003	1.615 (1.183-2.205)	0.648	0.651

*OR* odds ratio, *NB-AUC* the area under the receiver operating characteristic curve (AUC) value by naïve Bayes, *LR-AUC* AUC value by logistic regression

**Table 3:** Association of hypertension with physical factors and predictive powers of individual factors in the Gwan position of the right wrist

Variable	Crude		Adjustment for age and BMI		Predictive power	
	p	OR	p	OR	NB-AUC	LR-AUC
R-PPI	<0.001	2.648 (1.909-3.672)	<0.001	2.504 (1.802-3.479)	0.759	0.763
R-PDI	0.073	1.294 (0.976-1.715)	0.097	1.277 (0.956-1.705)	0.521	0.537
R-Sum-30p	<0.001	1.787 (1.37-2.33)	<0.001	1.776 (1.354-2.329)	0.627	0.652
R-Sum-30p-PPI	<0.001	1.89 (1.45-2.464)	<0.001	1.891 (1.436-2.49)	0.611	0.655
R-Sum-1.12v	<0.001	1.861 (1.426-2.428)	<0.001	1.845 (1.406-2.422)	0.639	0.666
R-Sum-1.12v-PPI	<0.001	2.001 (1.531-2.616)	<0.001	1.997 (1.513-2.637)	0.63	0.673
R-Asys-HR75	0.233	1.196 (0.891-1.606)	0.158	1.242 (0.919-1.679)	0.523	0.545
R-PSD-w1	<0.001	1.76 (1.288-2.405)	0.001	1.677 (1.226-2.295)	0.635	0.638
R-PSD-w2	0.013	1.425 (1.076-1.887)	0.056	1.322 (0.992-1.761)	0.564	0.575
R-PSD-0-13Hz	<0.001	1.885 (1.381-2.573)	<0.001	1.782 (1.299-2.444)	0.67	0.671

*OR* odds ratio, *NB-AUC* the area under the receiver operating characteristic curve (AUC) value by naïve Bayes, *LR-AUC* AUC value by logistic regression

### Comparison of the predictive power of the individual and combined physical factors

Prediction methods using a combination of physical factors based on two machine learning algorithms consisted of wrapper-based feature selection with NB (Wrapper: NB), wrapper-based feature selection with LR (Wrapper: LR), and LASSO with LR (LASSO: LR).

Table 4 provides a comparison of the predictive powers of the combined physical factors for the diagnosis of hypertension. The predictive power of the combined physical factors of the right wrist was higher than that of the left wrist, similar to the analysis of the individual physical factors. Compared with the single best predictor (L-PPI) in the left wrist, models using Wrapper: NB and Wrapper: LR exhibited a slight improvement in AUCs of 0.043 for NB and 0.045 for LR. In comparison with the single strongest indicator (R-PPI) in the right wrist, models using the two methods exhibited a slight improvement in AUCs of 0.02 for NB and 0.015 for LR. In the two machine-learning algorithms, the predictive power of the Wrapper: LR method was slightly better than that of Wrapper: NB for the left wrist, but the powers of Wrapper: NB and Wrapper: LR were approximately equal for the right wrist. In all experiments, although the BASSO: LR method obtained the lowest predictive power, the method may solve the multicollinearity.

**Table 4:** Analysis of the predictive powers of combined physical factors for hypertension diagnosis

Wrist	Method	Status	AUC	Sensitivity	1-specificity	Precision	F-measure
Left	Wrapper: NB	Normotension	0.735	0.977	0.846	0.883	0.928
		Hypertension		0.154	0.023	0.5	0.235
	Wrapper: LR	Normotension	0.745	0.988	0.942	0.873	0.927
		Hypertension		0.058	0.012	0.429	0.102
	LASSO: LR	Normotension	0.722	0.985	0.885	0.880	0.929
		Hypertension		0.115	0.015	0.545	0.190
Right	Wrapper: NB	Normotension	0.779	0.968	0.788	0.889	0.927
		Hypertension		0.212	0.032	0.5	0.297
	Wrapper: LR	Normotension	0.778	0.979	0.846	0.884	0.929
		Hypertension		0.154	0.021	0.533	0.239
	LASSO: LR	Normotension	0.757	0.977	0.827	0.886	0.929
		Hypertension		0.173	0.023	0.529	0.261

AUC: area under the receiver operating characteristic curve; Wrapper: NB, wrapper-based feature selection method with naïve Bayes; Wrapper: LR, wrapper-based feature selection method with logistic regression; LASSO: LR, the least absolute shrinkage and selection operator with logistic regression

For the variable selection methods, the numbers of selected variables ranged from 2 to 8 physical factors. For example, the model built using Wrapper: LR for the left wrist included 5 physical factors: L-PPI, L-Sum-30p, L-Sum-30p-PPI, L-Asys-HR75, and L-PSD-0-13Hz. These selected physical factors in each model differed due to the characteristics of the wrapper-based variable selection with the two machine learning algorithms and the LASSO with LR algorithm. Table 4 presents the detailed results of each prediction model using combined physical factors, and Table 5 presents the physical factors inserted in each final model using the wrapper-based variable selection and LASSO techniques. The predictive power of using combined physical factors was higher than that of the best single predictors for both the left and right wrists. Thus, a combination of physical factors based on computational methods and data mining are useful for improved diagnosis of hypertension using the wrist pulse wave.

**Table 5:** Selected physical factors in both the left and right wrists using variable selection techniques

Wrist	Wrapper: NB	Wrapper: LR	LASSO: LR
Left	L-PPI, L-PSD-0-13Hz	L-PPI, L-Sum-30p, L-Sum-30p-PPI, L-Asys-HR75, L-PSD-0-13Hz	L-PPI, L-PDI, L-Sum-30p-PPI, L-Sum-1.12v, L-Asys-HR75, L-PSD-w1, L-PSD-w2, L-PSD-0-13Hz
Right	R-PPI, R-PDI, R-PSD-0-13Hz	R-PPI, R-PSD-0-13Hz	R-PPI, R-PDI, R-Sum-1.12v, R-Sum-1.12v-PPI, R-PSD-w1, R-PSD-0-13Hz

Wrapper: NB, wrapper-based feature selection method with naïve Bayes; Wrapper: LR, wrapper-based feature selection method with logistic regression; LASSO: LR, the least absolute shrinkage and selection operator with logistic regression

## Discussion

In this study, we demonstrated that PPI was the factor most strongly associated with hypertension and the best indicator in both wrists. Physical factors in the right wrist tended to have higher predictive power than in the left wrist, and combined physical factors yielded better predictive power compared with single physical factors in both wrists.

Many studies of TCM and TKM have attempted to obtain scientific evidence of an association between the wrist pulse wave and internal organs or diseases based on Oriental medicine theory or physiologic mechanisms [3, 6, 21, 30], in addition to constitutional diagnosis [31, 32]. In general, these studies have focused on the standardization and quantification of pulse patterns and associations between pulse patterns and diseases [2, 3, 11–14]. For the prediction of hypertension based on TCM pulse diagnosis, Tang et al. [3] examined diagnostic models of hypertension using an artificial neural network based on 8 elements (i.e., depth, strength, stiffness, rate, regularity, smoothness, width, and length) in both the left and right wrists in the normal subject group and the hypertension group. They demonstrated the prediction of hypertension using features from the wrist pulse in TCM with accuracies of approximately 73 % to 79 %. There are several differences between our study and Tang et al. [3] for predicting hypertension using the TCM pulse wave. First, the study by Tang et al. [3] used only 8 features (i.e., depth, strength, stiffness, rate, regularity, smoothness, width, and length), whereas we used the physical factors extracted from the wrist pulse waves. Second, while the numbers of negative and positive samples were similar in the previous study, the number of hypertensive samples in our study was less than that of normal subject samples, resulting in a class imbalance problem. The large difference in the sizes of the negative and positive samples can result in an unbalanced class problem and may lead to biased results that tend to have high predictive power toward many sample sizes and low power toward small sample sizes because of the characteristics of traditional classification or prediction algorithms [33–38]. Finally, Tang et al. [3] used different criteria for normotension than those used in our study. Despite these differences, we agree with the results of Tang et al. [3] indicating that it is possible to predict hypertension based on the pulse diagnosis, and these studies support the quantification and standardization of pulse patterns.

Certain investigators have reported the influence of arm dominance according to gender. Rogers et al. [39] argued that in women, “the pulses should be a little stronger on the right wrist, but pulses in men should be stronger on the left”. King et al. [40] examined the differences in depth, overall and relative pulse force, width, and rhythm at three sites between healthy male and female participants. They reported that the pulse force of men was stronger than that of women. Furthermore, they reported that the mean systolic and diastolic BPs were significantly lower in women than in men and that the mean SBP was significantly higher in the right wrist than the left wrist in both men and women. King et al. [41] documented that, out of three possible states with regard to the dominant side (right, left, and neither), the pulse in the right wrist of right-handed subjects was generally stronger than those in the left wrist of left-handed subjects or in either wrist of subjects in whom neither arm was dominant. Furthermore, they mentioned that the difference in systolic pressure and pulse amplitude in the right arm was higher than that in the left arm in the cohort study. These findings are consistent with our results, which indicated that the right wrist had a higher association with BP compared with the left wrist in women. It is important to consider the association between BP and arm dominance. However, we did not consider the effect of right- or left-handedness in this study. We may assume that the association of physical factors from the right wrist was higher than that from the left wrist because the majority of the Korean population is right handed. Further study is necessary to reveal an association between differences in right and left BPs and the dominance of the arm.

To obtain scientific evidence for pulse diagnosis and the association between wrist pulse patterns and diseases, several recent studies have employed computational approaches [5, 7, 10, 11, 21, 30, 42, 43]. For example, Dongyu et al. [11] examined the prediction of diagnosis in gastritis and cholecystitis patients compared with healthy subjects using features extracted from wrist pulse signals. Chen et al. [21] classified a healthy group and patient groups with duodenal bulb ulcers and pancreatitis based on variables extracted from pulse waves. Furthermore, Shin et al. [5] suggested a regression equation using 15 multiple parameters to enhance the accuracy of BP measurement based on a pulse diagnostic apparatus widely used in TKM and TCM and developed an Android application for use with a pulse diagnostic apparatus device for BP measurement. Lukman et al. [10] reviewed data mining techniques, databases, expert systems, and biomedical mining systems to evaluate TCM practices and herbs such as wrist pulse diagnosis and other TCM diagnoses. They also argued that the computational approach was valuable for discovering future medical knowledge in TCM. Kim et al. [42] examined the association between eating time and blood circulation and pulse wave energy in the Chon, Gwan, and Cheok positions of both wrists in healthy adult men based on computational analysis. They observed an increase in the pulse wave energy after meal consumption, followed by gradual decreases at all positions of both wrists. They suggested that eating and differences in eating times may lead to changes in the pulse wave energy and the blood circulation index in the pulse wave. Ferreira [30] explored the association between internal organs and compression and amplification factors from wrist pulse waves based on resonance theory using a computational simulation incorporating a stochastic procedure. Wrist pulse waves were extracted in the Chon, Gwan, and Cheok palpation positions and obtained by the “pressing with one finger” method. They argued that certain pulse wave harmonics could be considered as a function of depth in the Chon, Gwan, and Cheok palpation sites by amplification. Kim et al. [7] reviewed wrist pulse diagnostic devices in terms of sensors, actuators for skin and pressure control, diagnosis systems, physical quantification, clinical studies, and the application of ubiquitous health systems in Korea and emphasized quantification and objectification of evidence-based medicine from medical data in TCM and TKM. Furthermore, supporting the hypothesis that pulse waves of the three wrist sites indicate different medical information in TCM or TKM, Jeon et al. [43] reported that the baseline and signal strengths differed statistically among the three wrist sites. These previous studies and our study demonstrate the importance of quantifying and objectifying pulse patterns and disease in TCM and TKM for evidence-based medicine based on a computational approach.

Finally, it is widely known that PPI is the difference between the systolic and diastolic pressures and is closely related to cardiovascular disease or hypertension [44]. Our results indicated that PPI was the best predictor of hypertension among all physical factors. In this study, we suggested new physical factors related to the sum of the area on the particular region of pulse waves in both wrists. The pattern of the radial artery pulse can appear in various forms: pulse amplitudes exhibit a gradual increase and decrease in some cases, whereas, in other cases, a rapid and sharp fluctuation occurs. A string-like pulse occurs when the arterial wall stiffens, indicating that the arterial wall resists deformation with increasing hold-down pressure [45]. The degree of the blood vessel resistance against the pressing force to the radial artery could be expressed as several factors measured by the KIOM pulse wave detector device, such as Sum-30p, Sum-30p-PPI, Sum-1.12v, and Sum-1.12v-PPI, because the blood vessel resistance has a tendency to increase the quantity of those factors. We were able to acquire physical factors related to the summation of the area on pulse waves by using the device adapting a continuously evolving tonometric mechanism. To date, no study has provided the analytical result of predictive powers using these physical factors.

### Limitations and future work

Although this pilot study demonstrated several significant findings, there were limitations. First, this study used physical factors extracted from only one of three positions. A comparison of the physical factors from the Chon and Cheok positions in addition to the Gwan position is necessary. Second, to predict hypertension more successfully, more reliable and stable devices must be developed for pulse measurement in the three positions in both the left and right wrists. Third, larger samples and further studies are required for evaluating the differences in hypertension and the physical factors of the wrist pulse waves between women and men. Finally, further investigation is necessary to reveal the association between differences in the right and left BP and the dominance of the arm.

## Conclusion

Pulse diagnosis and patterns comprise one of the core research areas in TCM and TKM. However, pulse diagnosis and patterns are based on subjective judgment and are influenced by the experience or intuition of the Oriental physician performing these procedures; they are also difficult to learn due to the lack of standardization and quantification of wrist pulse and patterns and the association between pulse patterns and diseases. The findings of this pilot study demonstrate that among the physical factors that were objectified and quantified by the characteristics of the wrist pulse patterns, PPI in both wrists was most strongly associated with hypertension and was the best indicator of hypertension. The predictive power of physical factors of the right wrist was higher than that of the left wrist. Furthermore, a combination of physical factors based on a computational method and data mining is useful for improved diagnosis of hypertension using the wrist pulse wave. The results and methods of this study may support the quantification and objectification of pulse patterns and diseases in TCM and TKM for complementary and alternative medicine based on a computational approach. This is the first study to report the predictive power of combined physical factors for identifying hypertension in Korean adult women.

## Abbreviations

TCM: Traditional Chinese medicine
TKM: Traditional Korean medicine
LR: Logistic regression
NB: Naïve Bayes algorithm
PPI: Maximum pulse amplitudes
AUC: The area under the receiver operating characteristic curve
BMI: Body mass index
SBP: Systolic blood pressure
DBP: Diastolic blood pressure
